# A novel strategy for orthogonal genetic regulation on different RNA targeted loci simultaneously

**DOI:** 10.1080/15476286.2022.2141507

**Published:** 2022-11-09

**Authors:** Yongjian Guo, Liang Wang, Zhen Qi, Yu Liu, Kun Tian, Huanran Qiang, Pei Wang, Guohua Zhou, Xiaobo Zhang, Shu Xu

**Affiliations:** a School of Basic Medical Science and Clinical Pharmacy, China Pharmaceutical University, Nanjing, 210000, China; b School of Biopharmacy, China Pharmaceutical University, Nanjing, 210000, China; c Institute of Binjiang, Zhejiang University, Hangzhou, 310053, China; d Women’s Hospital of Nanjing Medical University, Nanjing Maternity and Child Health Care Hospital, Nanjing, 210000, China; e Department of Pharmacology, Jinling Hospital, Medical School, Nanjing University, Nanjing, 210000, China

**Keywords:** Orthogonal genetic regulating, mRNA, miRNA, FEN1, mis-hpDNA

## Abstract

No current RNA-targeted interference tools have been reported to simultaneously up and down-regulate different gene expressions. Here we characterized an RNA-targeted genetic regulatory strategy composed of a flap endonuclease 1 (FEN1) and specific mis-hairpin DNA probes (mis-hpDNA), to realize the orthogonal genetic regulation. By targeting mRNA, the strategy hindered the translation and silenced genes in human cells with efficiencies of ~60%. By targeting miRNA, the strategy prevented the combination of miRNA to its specific mRNA and increased this mRNA expression by about 3-folds. In combination, we simultaneously performed *CXCR4* gene knock-down (~50%) and *EGFR* gene activation (1.5-folds) in human cells. Although the functional property can be further improved, this RNA-targeted orthogonal genetic regulating strategy is complementary to classical tools.

## Introduction

Present RNA-level interference tools are siRNAs [1], antisense oligonucleotides (ASOs) [2], RNA-targeted CRISPR/Cas systems [3–6], miRNA mimetics [7], antimiRs [8] and so on. Up to now, no individual tool has been reported to be capable of simultaneously up and down-regulating gene expression when targeting RNA loci. In fact, in some cases, researchers need to simultaneously work on different splice isoforms and regulatory RNA elements, or even up-regulate and down-regulate the expression of different genes simultaneously to characterize gene functions comprehensively. Traditionally, it requires various tools working in the same cell, leading to inconvenience in operation and maybe mutual interference in effect.

**Figure 1.**
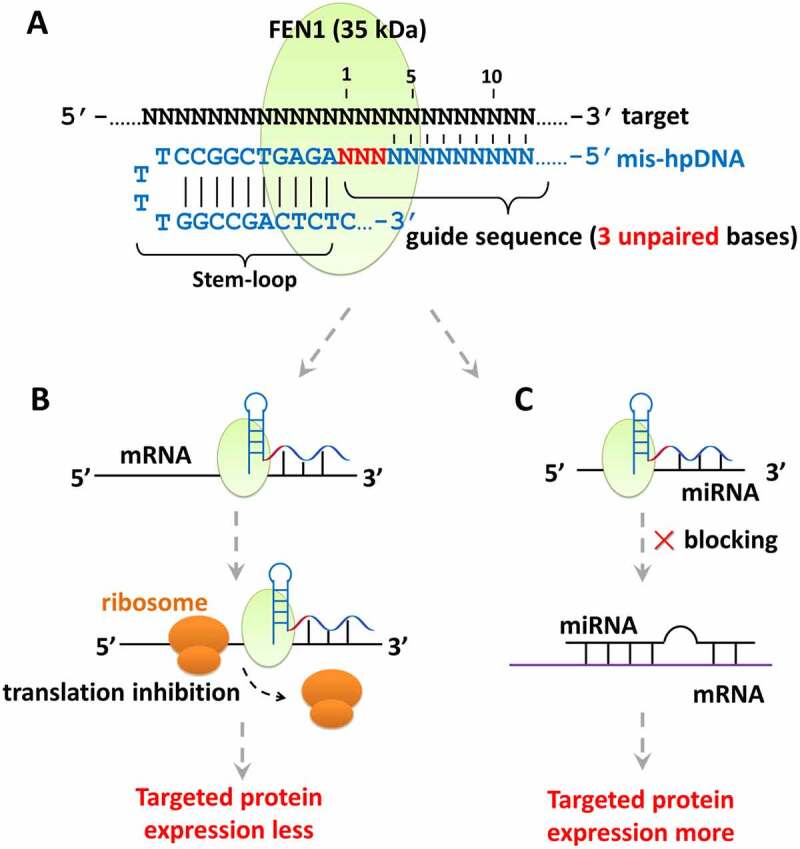
**Orthogonal genetic regulation on different targeted RNA loci**. (A) The RNA-targeted strategy composes a FEN1 nuclease (~35 kDa) and specific mis-hpDNA probes (including stem-loop and guide sequence). There are 3 unpaired and more than 15 paired bases in the guide sequence. (B) The FEN1-probe complex blocks the moving forwards of the ribosome to inhibit the mRNA translation and down-regulate gene expression. (C) FEN1-probe complex blocks miRNAs to form the miRNA:mRNA complex and makes the free mRNA more to up-regulate gene expression.

In this study, we designed an RNA-targeted strategy composed of a Flap Endonuclease-1 (FEN1, ~35 kDa) [9] and specific mis-hpDNA probes (including stem-loop and guide sequence), to orthogonal regulate gene expression (Figure 1A). FEN1 captures the mis-hpDNA probe due to its stem-loop structure [10,11]. Then this FEN1-probe complex locates on the target loci, which is guided by the guide sequence in the mis-hpDNA probe, and makes a steric hindrance effect on the targeted mRNA or miRNA. The FEN1-probe complex further blocks the moving forwards of the ribosome to inhibit the mRNA translation to down-regulate gene expression (Figure 1B), or blocks miRNAs from forming miRNA:mRNA [12,13] complexes and makes the free mRNA more to up-regulate gene expression (Figure 1C). We named this strategy as ***hp****DNA-assisted**s**tructure-**g**uided**n**uclease mediating **i**nterference* (designated the **HpSGNi** system hereafter) and tested the loss/gain function separately and together here.

## Result

### 1. Targeting mRNA to mediate gene silencing in human cells.

**Figure 2.**
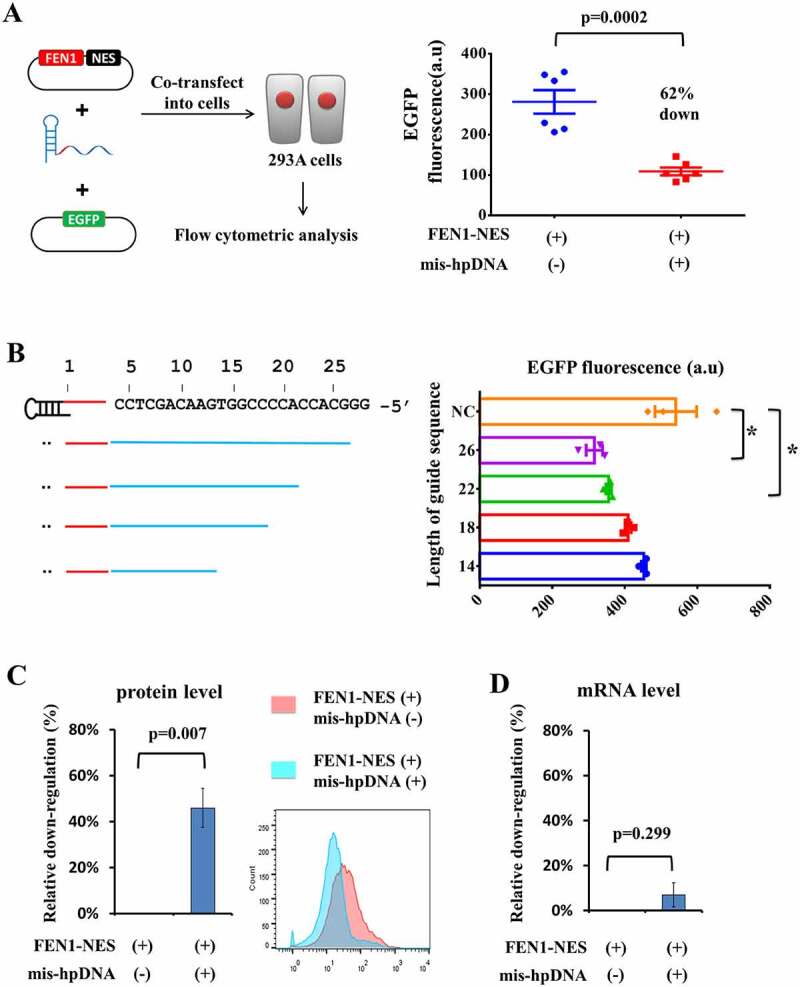
**Targeting mRNA to mediate gene silencing in human cells**. (A) Schematic diagram of EGFP down-regulation in HEK293A cells heterologously expressing FEN1-NES. EGFP fluorescence in HEK293 cells transfected with FEN1-NES plus specific mis-hpDNA or not. mean ± SEM. n = 6. (B) Sensitivity of the guide sequence length. NC: the group was transfected with unrelated probes. mean ± SEM. n = 3. * p < 0.05. (C) Relative CXCR4 expression of protein level and (D) mRNA level in A549 cells transfected with FEN1-NES plus specific mis-hpDNA or not. mean ± SEM. n = 3.

Firstly, we tested whether we could target mRNA and mediate gene silencing in human cells. HEK293A cells were co-transfected with plasmids encoding FEN1-NES (an NES was into the C terminal of FEN1, which made the protein expressed only in the cytoplasm, not in the nucleus, Figure S1), plasmids encoding EGFP, and mis-hpDNAs targeting the *EGFP* mRNA gene (Figure 2A).

Then the fluorescence in cells was detected by flow cytometric analysis, and the efficiency of down-regulation of the *EGFP* gene was calculated by comparing with control groups. As shown in Figure 2A and Figure S2, the normalized fluorescence of the group transfected with mis-hpDNA plus FEN1-NES was 62% lower on average than that in the group transfected with unrelated probes, with a significant difference (p-value was 0.0002). To know the effect of the guide sequence length on down-regulation efficiency, we shorten the length from 26-nt to 14-nt with a 4-nt interval (Figure 2B). For mis-hpDNA with 26- and 22-nt guide sequences, the EGFP expression was reduced obviously (p-value were 0.02 and 0.03, respectively). But for 18- and 14-nt guide sequence, the reduction was no longer obvious (p-value were 0.08 and 0.20, respectively). It indicated that too short guide sequences with relatively low Tm values are not beneficial for binding with targeted RNA loci. So, we suggested the guide sequence should not be too short, more than 22-nt.

Secondly, we co-transfected A549 cells with plasmids encoding FEN1-NES and mis-hpDNAs targeting the *CXCR4* mRNA. Compared with the control groups that were transfected with FEN1-NES alone, certain (~45%) down-regulation of CXCR4 expression in the group transfected with both FEN1-NES and specific mis-hpDNA was observed (Figure 2C). The location of the mis-hpDNA for *CXCR4* mRNA crossed two exons in genomic DNA, and the knockdown cannot be attributed to DNA-level transcription blocking. Then, we quantified the *CXCR4* mRNA to see whether it was cleaved or not. The *CXCR4* mRNA level in the group transfected with FEN1-NES plus specific mis-hpDNA shows an indistinctive difference from that in the group transfected with FEN1-NES alone (Figure 2D). Therefore, the knockdown of CXCR4 expression cannot be attributed to mRNA cleavage. Thus, it was concluded that the FEN1-probe complex can be reprogrammed to preferably mediate gene silencing by blocking but not cleaving mRNA in human cells.

## 2. Targeting miRNA to mediate specific gene up-regulating in human cells

**Figure 3.**
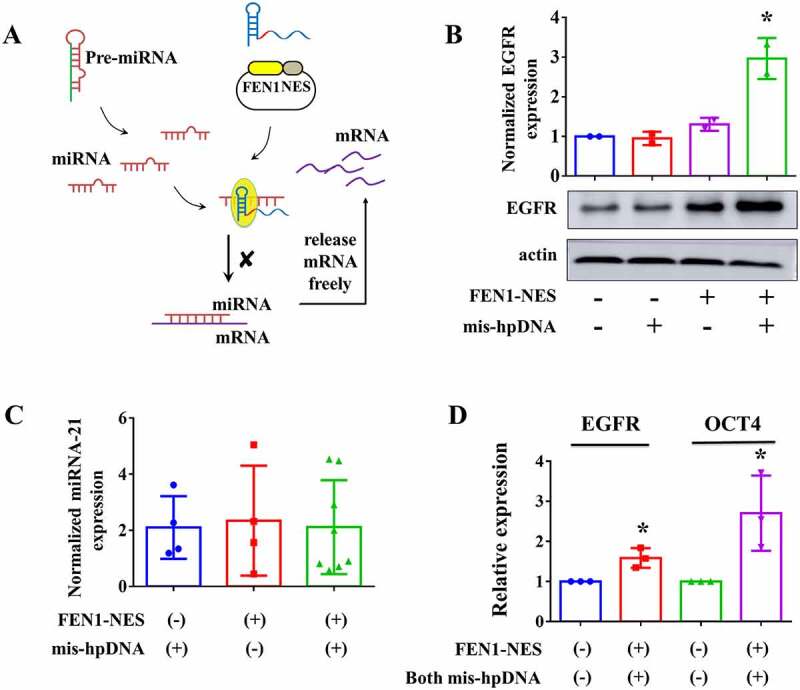
**Targeting miRNA to mediate specific gene up-regulating in human cells**. (A) Schematic diagram of HpSGNi guided up-regulation in A549 cells heterologously expressing NES-FEN1. (B) The relative expression of EGFR in A549 cells transfected with FEN1-NES plus mis-hpDNA or not. mean ± SD. n = 2. * p value < 0.05. (C) The normalized expression of miRNA-21 in A549 cells transfected with FEN1-NES plus mis-hpDNA or not. mean ± SEM. n = 3 to 8. (D) The relative expression of EGFR and OCT4 in A549 cells transfected with FEN1-NES plus mis-hpDNA or not. n = 3. mean ± SD. * p value < 0.05.

After verifying the ability of down-regulating gene expression, we then test whether it can also up-regulate in human cells. The miRNA [12] was reported with the ability to capture the specific mRNA and guide degradation/blocking. In this study, as shown in Figure 3A, we tried to use FEN1 plus mis-hpDNA to capture the miRNA, then inhibit the combination between miRNA and its targeted mRNA, followed by holding back the mRNA degradation/blocking.

In studies reporting the binding ability of miRNA and targeted RNA [14], miRNA-21-5p has been reported in relationship with regulating EGFR [15]. FEN1-NES plasmid was co-transfected with mis-hpDNA targeting miRNA-21-5p. As Figure 3B showed, the group transfected with the FEN1-NES plus mis-hpDNA increased EGFR protein expression (best ~ 3-folds) than the group transfected with FEN1-NES alone. To confirm that HpSGNi did not cleave targeted miRNA, we used quantitative PCR to quantify the amount of miRNA-21-5p. Compared with control groups, no depletion in experimental groups was observed (Figure 3C).

Then we further test whether multiple miRNAs could be blocked. The miRNA-145-5p [16] was reported to be capable of targeting *OCT4* (also named *POU5F1P1*) mRNA. We transfected mis-hpDNAs targeting miRNA-145-5p and miRNA-21-5p into A549 cells expressing FEN1-NES. Results in Figure 3D showed that both EGFR and OCT4 expression were up-regulated (~1.5 and ~2.5-folds on average, respectively). Thus, it was concluded that the FEN1-probe complex could be reprogrammed to preferably mediate gene up-regulation by targeting miRNA in human cells. However, we worry that the efficiency will decrease when the number of targeted miRNA increases. It was confirmed when we transfected three kinds of mis-hpDNAs to up-regulate both OCT4, EGFR and YWHAZ expression. One of the reasons is that the FEN1-NES will be shared with different targeted loci. Another reason is the amount of each mis-hpDNA will be decreased when the number of kinds of mis-hpDNA increases. The ability to up-regulate many genes simultaneously still needs to be improved in future studies.

## 3. Orthogonal gene down- and up-regulation simultaneously

**Figure 4.**
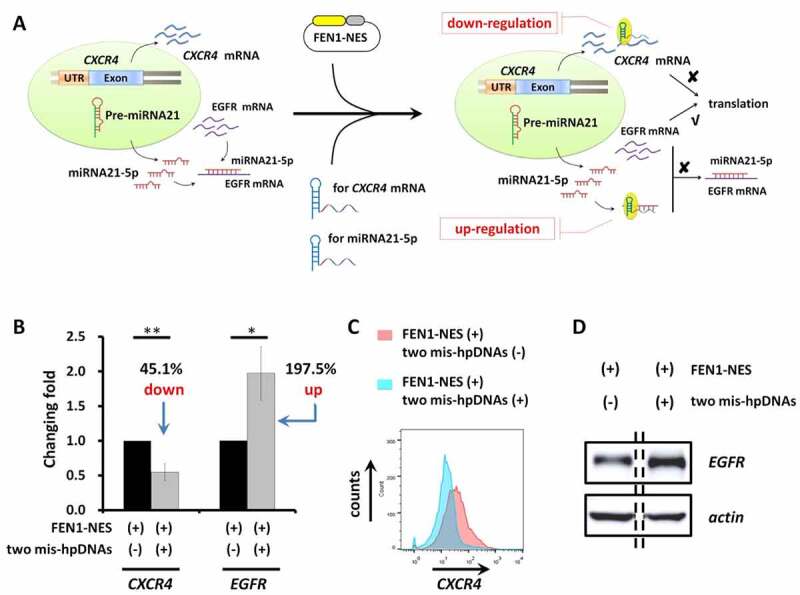
Orthogonal gene down/up-regulation simultaneously. (A) Schematic diagram of HpSGNi guided orthogonal gene down- and up-regulation in A549 cells simultaneously. (B) The change of expression of CXCR4 and EGFR in protein level in A549 cells transfected with specific mis-hpDNA or not. n = 3. mean ± SD. * p value < 0.05. ** p value < 0.01. (C) A typical flow cytometric analysis of CXCR4 in A549 cells transfected with specific mis-hpDNA or not. (D) A typical WB result of EGFR in A549 cells transfected with specific mis-hpDNA or not.

To explore whether one gene expression could be suppressed and another gene expression could be active simultaneously, we mediate orthogonal gene control (down- and up-regulation) using the FEN1-NES, a mis-hpDNA targeting *CXCR4* mRNA and a mis-hpDNA targeting miRNA-21-5p (Figure 4A). A549 cells were transfected with plasmids encoding FEN1-NES, then were transfected with mis-hpDNA targeting miRNA-21-5p for *EGFR* gene activation and mis-hpDNA targeting *CXCR4* mRNA for *CXCR4* gene silencing. As a result, CXCR4 protein expression decreased by~50% (Figure 4B, and C), and EGFR protein expression increased by about 2-folds (Figure 4B, and D) in this orthogonal condition.

## Discussion

FEN1 is a nuclease being able to recognize and cleave nucleic acid. During DNA replication and repair processes, the newly synthesized DNA and the displaced region compete for base pairing with the template strand, forming a double-flap structure [17]. The FEN1 recognizes the double-flap structure by the 3’ flap and catalyzes phosphodiester cleavage of the 5’ flap [11]. Then FEN1 participates in removing RNA primers. Then in 2020, we used the characteristic of FEN1 to build a ***h**air**p**in DNA probe **s**tructure-**g**uided**n**uclease* system (designated the **HpSGN** system hereafter) [18]. The HpSGN system comprises FEN1 nuclease and hairpin DNA probe (designated hpDNA below). Significantly, a notable feature is that the cleavage is invalid when the base at the position of 1 on the target mismatches the hpDNA [18]. We inferred that this feature could be taken to build an interference platform. And to confirm this point, we mutated the bases in the hpDNA at positions of 1–3, followed by reacting with single-strand RNA targets (ssRNA) and FEN1. As the electrophoretic mobility shift assay (EMSA) in Figure S3 shows, the mis-hpDNA frustrated FEN1 cleavage but formed a FEN1-hpDNA-target ternary complex. These observations suggested that we can build the interference platform HpSGNi, which can capture ssRNA targets without cleavage.

To our knowledge, HpSGNi was the first reported RNA-targeted loss and gain-function methodology. But at present, the number of genes that can be regulated simultaneously is limited by transfection and cell toxicity (Figure S4). And when compared separately, this orthogonal genetic regulation system has lower efficiency of up-regulation than ORFs and does not perform better than RNAi and CRISPRi to down-regulate.

However, the HpSGNi system still has its own advantages. It has no limitation on targets’ sequences (like PFS for RNA-targeted CRISPR/Cas system) and is theoretically suitable for RNA targets with any sequence. In contrast with the Cas proteins, which were 1367 to 422 amino acids, the FEN1 protein is 337 amino acids. The smaller size of the FEN1 is of great benefit to delivery. It has no need to be chemically modified, which makes the strategy cheaper than synthetic oligonucleotides. Theoretically, the stability of the DNA probe is better than the RNA probe, which may be one of the advantages of HpSGNi system. So, we modified a Cy5 label on the mis-hpDNA and transfected it into cells to observe after 12, 24 and 48 hours. As shown in Figure S5, the mis-hpDNA remained stable after being transfected into cells 48 hours later. Compared with other endogenous miRNAa strategies [19], the mechanism of HpSGNi is relatively simple and relies on a fixed FEN1:mis-hpDNA:miRNA complex. But this kind of miRNAa methods may be partly affected by the cell type and targeted miRNA type, for example, with no strong miRNA /mRNA interaction, there will be no mRNA up-regulation

In summary, we developed a simple and modular RNA-targeted platform HpSGNi system for targeted gene regulation. Although the efficiency should be improved in further study and the compatibility of application on the miRNA was limited, the strategy reported here is still complementary to classical methods.

## Materials and methods

### Construction of plasmid expressing FEN1

For RNA loci regulating in cells, NES was added to the C-terminus of synthesized *A. fulgidus* FEN1 ORF and subcloned to pcDNA3.1(+) to generate pcDNA3.1(+)-HA-*A. fulgidus* FEN1-NES (Figure S1).

### Designing the mis-hpDNAs

The mis-hpDNA consisted of a stem-loop and a guide sequence. The stem-loop was fixed as *5’-aga gtc ggc ctt ttg gcc gac tct ctt atc aac ttg aaa aag ttg gca ccg agt cgg tgt-3’*. The guide sequence is shown in Table 1. The unpaired bases are marked in red.

**Table 1.** The guide sequence of mis-hpDNA used in this study.

Name	Sequence (5’ to 3’)
mis-hp-CXCR4	ACTGATCCCCTCCATGGTAACCGCTcca
mis-hp-EGFP	GGGCACCACCCCGGTGAACAGCTCCcgt
mis-hp-EGFP-26	GCACCACCCCGGTGAACAGCTCCcgt
mis-hp-EGFP-22	CACCCCGGTGAACAGCTCCcgt
mis-hp-EGFP-18	CCGGTGAACAGCTCCcgt
mis-hp-EGFP-14	TGAACAGCTCCcgt
mis-hp-mi21-5p	TCAACATCAGTCTGATAAGgat
mis-hp-mi145-5p	AGGGATTCCTGGGAAAACacc

### Cell culture and transfection

HEK293A and A549 cells were maintained in Dulbecco’s modified Eagle’s Medium (DMEM) or RPMI-1640 supplemented with 10% foetal bovine serum (HyClone), 100 U/mL penicillin, and 100 µg/mL streptomycin at 37°C with 5% CO_2_ incubation. Cells were seeded into 6-well plates/24-well plates (Corning) 24 h before transfection at a density of 300,000 /70,000 cells per well. Cells were transfected using GenJet™ Reagent (SignaGen) following the manufacturer’s recommended protocol. For each well of a 6-well/24-well plate, 1 μg/0.5 μg plasmids expressing FEN1 were transfected and 500 pmoL/100 pmoL of DNA probes were transfected 24 h later.

### Target mRNA prediction for miRNA

The targets of miR-145 and miR-21 were predicted by Miranda (http://www.miRBase.org).

### Western blot (WB)

Protein samples were isolated with lysis buffer, eluted with SDS buffer, separated by SDS-polyacrylamide gels, and electroblotted onto NC membranes. The specific protein bands were stained with High-sig ECL Western Blotting Substrate (Tanon) and imaged using the Amersham Imager 600 (GE Healthcare). Primary antibodies included antibodies against OCT4 (Abcam, ab200834, 1:10,000), EGFR (Abclonal Technology, A11577, 1:1000) and β-actin (Abclonal Technology, AC026, 1:50,000).

### Flow cytometric analysis

The cells were resuspended in PBS, and the fluorescence intensity (EGFP 488 nm excitation and 525 nm emission) was measured immediately using FACSCalibur (Becton Dickinson). For CXCR4, cells were dissociated and then stained in PBS for 1 h at room temperature, followed by incubating with antibody (Miltenyi Biotec, 130–120-778, 1:50) and measured using FACSCalibur.

### Quantitative PCR

Eukaryotic cells were trypsinized and washed once with PBS, and total RNA was isolated with RNA-easy Isolation Reagent (Vazyme, R701-01/02) following the manufacturer’s instructions. For mRNA and miRNA, cDNA synthesis was performed using the HiScript III 1st Strand cDNA Synthesis Kit (+gDNA wiper) (Vazyme, R312-01) and 1st Strand cDNA Synthesis Kit (by stem-loop) (vazyme-MR101-01), respectively. The cDNA was used in quantitative PCR analyses with AceQ qPCR SYBR Green Master Mix (Low ROX Premixed) (Vazyme, Q131-02) and Universal SYBR qPCR Master Mix (Vazyme-MR101-01). Relative gene expression was calculated using the ΔΔCt method. Results were normalized to *Gapdh* or *U6* for mRNA and miRNA experiments. The primers used in this study are shown in Table 2.

**Table 2.** The qPCR primers used in this study.

Name	Sequence (5’ to 3’)
Gapdh-F	TAGTGGAAGGACTCATGACC
Gapdh-R	TCCACCACCCTGTTGCTGTA
CXCR4-F	GAAGCTGTTGGCTGAAAAGG
CXCR4-R	CTCACTGACGTTGGCAAAGA
miRNA-21-RT	GTCGTATCCAGTGCAGGGTCCGAGGTATTCGCACTGGATACGACTCAACA
miRNA-21-F	GCGCGTAGCTTATCAGACTGA
miRNA-21-R	AGTGCAGGGTCCGAGGTATT
U6-RT/U6-R	AACGCTTCACGAATTTGCGT
U6-F	CTCGCTTCGGCAGCACA

### Statistical analysis

All data were expressed as mean ± SD or mean ± SEM from two to eight independent experiments performed in a parallel manner. Comparisons between two groups were analysed using two-tailed Student’s t-tests. P values < 0.05 were considered statistically significant.

## Supplementary Material

Supplemental Material

## Data Availability

All data generated or analysed during this study are included in this article. The plasmids used in this study can be obtained from the corresponding authors upon request.
