# Supplementary material for: A novel strategy for orthogonal genetic regulation on different RNA targeted loci simultaneously
Source: RNA Biol. 2022 Nov 9;19(1):1172–8. doi: 10.1080/15476286.2022.2141507 (PMC9648401; doi:10.1080/15476286.2022.2141507)

SUPPLEMENTAL INFORMATION

**Figure S1. The detailed information of the plasmid pcDNA3.1(+)-HA-*A. fulgidus* FEN1-NES (The coding sequence of FEN1 was underlined and the coding sequence of NES was shown in italics).**


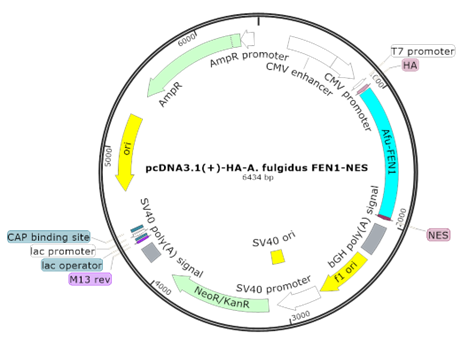


ATGGGTGCGGATATTGGTGACCTCTTTGAGAGGGAAGAGGTCGAGCTTGAGTACTTCTCAGGAAAGAAAATTGCCGTTGATGCTTTCAACACGCTATACCAGTTCATCTCGATAATAAGGCAGCCTGACGGTACGCCGTTAAAGGACTCACAGGGCAGAATCACCTCTCACCTTTCCGGAATCCTATACAGAGTCTCCAACATGGTCGAGGTGGGAATCAGGCCGGTGTTTGTATTCGACGGAGAGCCACCGGAGTTCAAGAAGGCTGAAATTGAGGAGAGGAAAAAGAGAAGGGCTGAGGCAGAGGAGATGTGGATTGCGGCTTTGCAGGCAGGAGATAAGGACGCGAAAAAGTATGCTCAGGCTGCAGGGAGGGTTGACGAGTACATTGTTGACTCCGCAAAGACGCTTTTAAGTTACATGGGGATTCCCTTTGTCGATGCCCCGTCTGAAGGAGAGGCGCAGGCTGCTTACATGGCAGCAAAAGGCGATGTGGAGTACACAGGAAGCCAGGATTACGATTCTCTGCTCTTCGGAAGCCCGAGACTCGCCAGAAATCTCGCAATAACGGGAAAAAGGAAGCTTCCCGGCAAAAATGTCTATGTGGATGTAAAGCCGGAGATAATAATTCTGGAAAGCAACCTCAAAAGGCTGGGTTTGACGAGGGAGCAGCTCATCGACATAGCGATTCTGGTCGGGACGGACTACAATGAGGGTGTGAAGGGTGTCGGCGTCAAGAAGGCTTTGAACTACATCAAGACCTACGGAGATATTTTCAGGGCACTCAAGGCTCTGAAAGTAAATATTGACCACGTAGAGGAGATAAGGAATTTCTTCCTGAATCCTCCTGTGACTGACGACTACAGAATAGAGTTCAGGGAGCCTGACTTTGAGAAGGCCATCGAGTTCCTGTGCGAGGAGCACGACTTCAGCAGGGAGAGGGTCGAGAAGGCCTTGGAGAAGCTCAAAGCTCTGAAGTCAACCCAGGCCACGCTTGAGAGGTGGTTC*CTGCCTCCACTTGAAAGACTGACACTG-*

**Figure S2. EGFP + cells percentage in HEK293 cells transfected with FEN1-NES plus specific mis-hpDNA or not.**

**
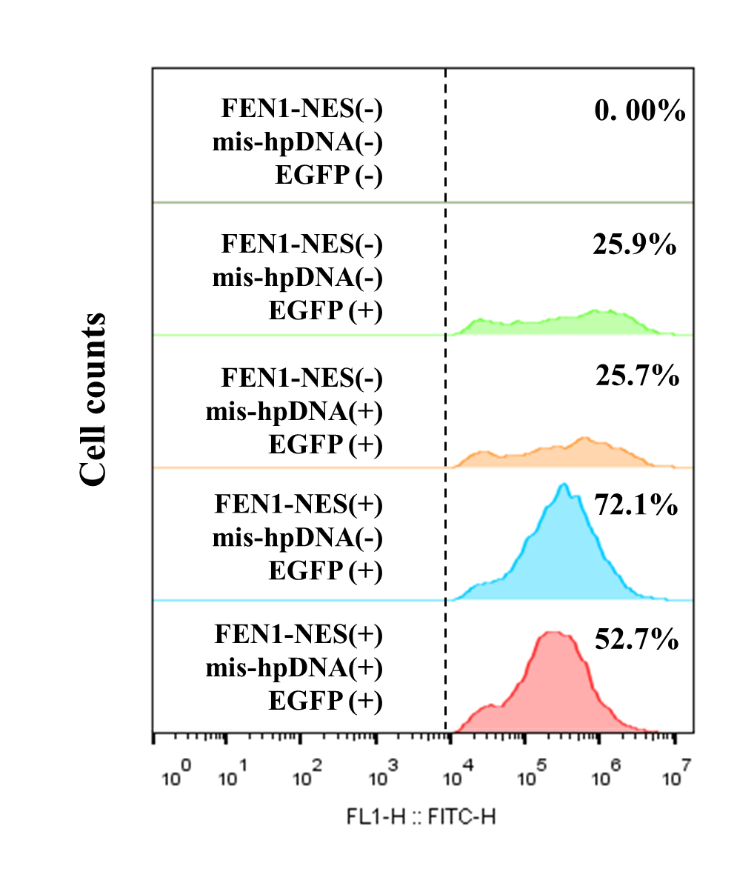
**

**Figure S3. The mis-hpDNA could frustrate FEN1 cleavage and form a FEN1-hpDNA-target ternary complex.**


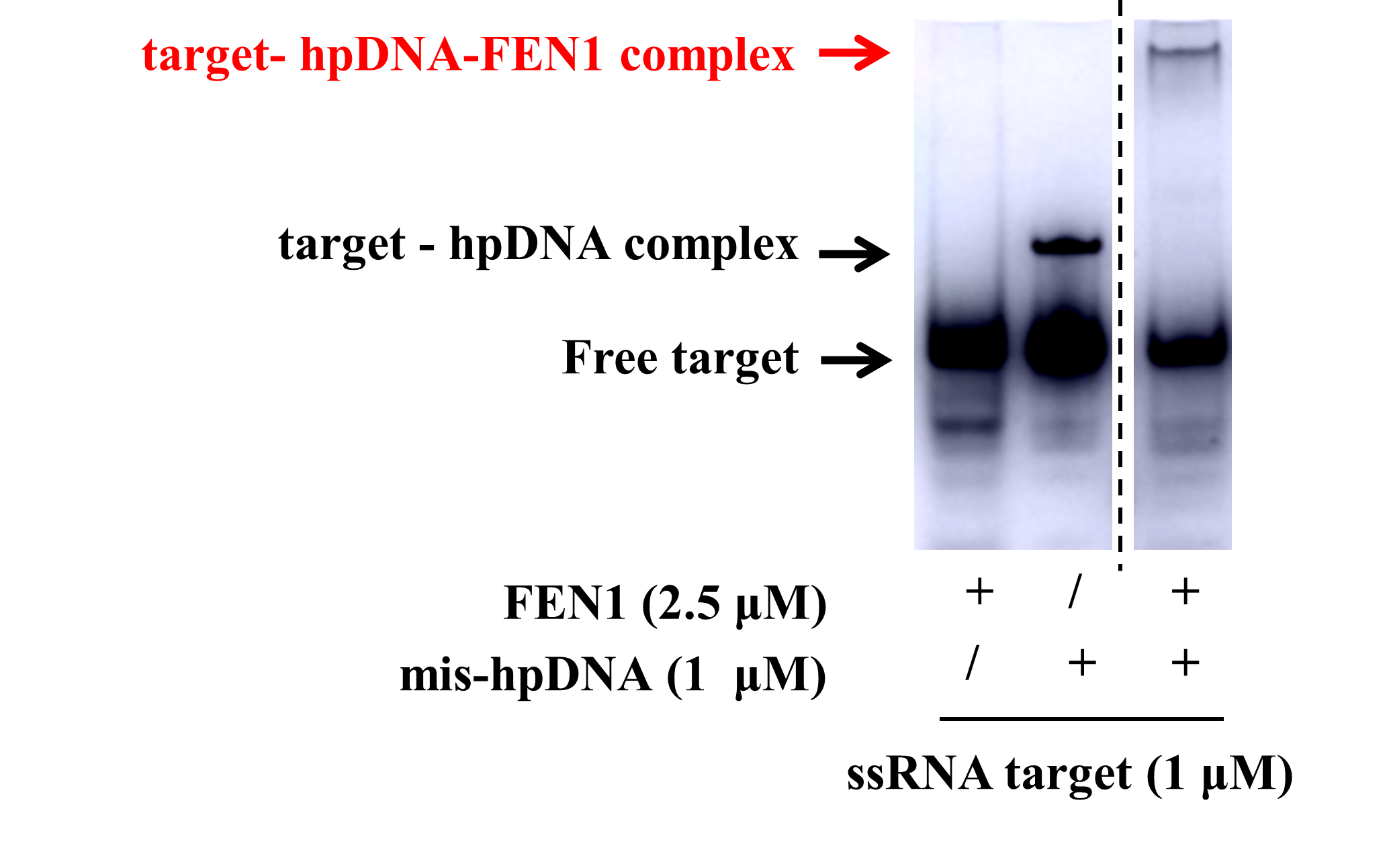


**Figure S4. HpSGNi have an effect on cell survival.**

**
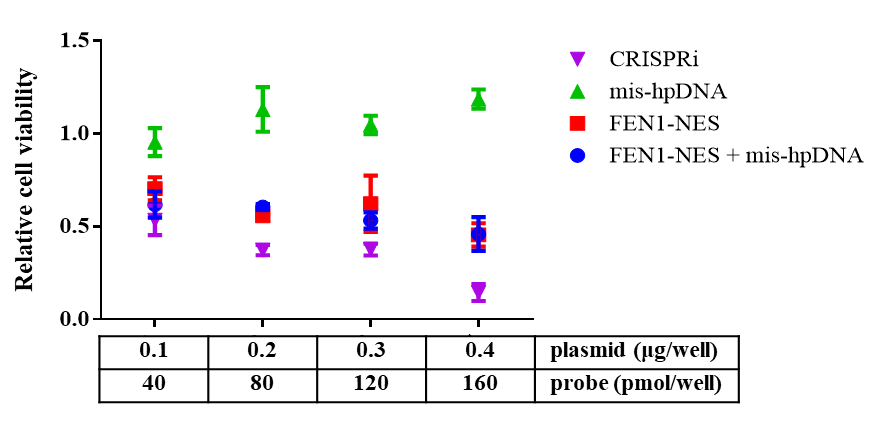
**

**Figure S5. The stability of mis-hpDNA in cells.**


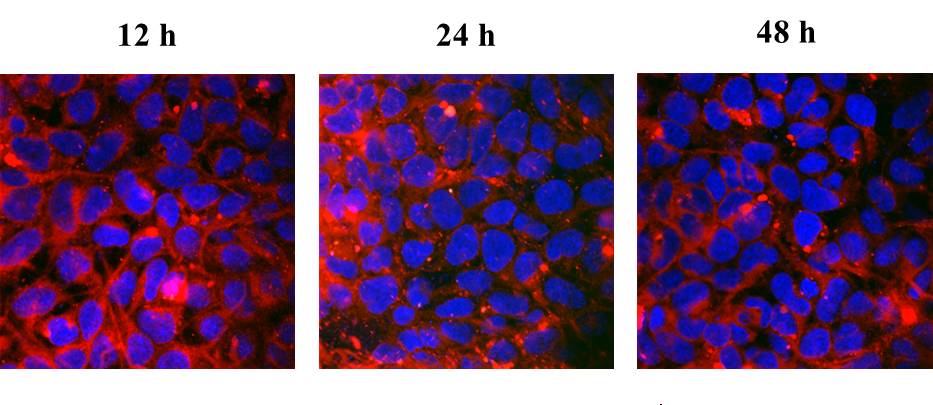

Supplement: Supplemental Material [file KRNB_A_2141507_SM9634.docx]
